# Educational nutritional intervention to prevent loss of health-related quality of life among older adults after a surgical treatment: design of a randomised controlled trial

**DOI:** 10.1186/s13063-024-08096-8

**Published:** 2024-04-15

**Authors:** Monica Christin Hansen, Lisbeth Uhrenfeldt, Kari Ingstad, Preben U. Pedersen

**Affiliations:** 1 Faculty of Nursing and Health Sciences, Nord University, Bodø, Norway; 2 Institute for Regional Health Research, Southern Danish University, Ortopedic dep., Lillebaelt University Hospital, Odense, Denmark; 3 Faculty of Nursing and Health Sciences, Nord University, Levanger, Norway; 4 Department of Clinical Medicine, Centre of Clinical Guidelines, Aalborg University, Aalborg, Denmark

**Keywords:** Disease-related malnutrition, Undernutrition, Older adults, Health-related quality of life, Clinical trial protocol, Instructional film and video

## Abstract

**Background:**

Disease-related malnutrition after a hospital stay has major consequences for older adults, the healthcare system and society. This study aims to develop and test the effectiveness of an educational video to prevent loss of health-related quality of life among live-at-home older adults after surgical treatment in a hospital.

**Method:**

This randomised controlled trial will occur at a regional hospital in Norway. Participants will be live-at-home adults aged 65 years and older. They will be recruited from three different surgical departments after a surgical procedure. Individuals with a body mass index below 24 and a home address in one of nine selected municipalities will be eligible for inclusion. Participants will be randomly assigned to either the intervention group or the control group. Those assigned to the intervention group will obtain access to a 6-min educational video 5 days after being discharged from the hospital. The control group will not obtain access to the video. The primary outcome will be health-related quality of life using the Norwegian Rand 36-Item Short Form Health Survey. Furthermore, we will measure body composition, number of readmissions and nutritional knowledge at inclusion and 3-month follow-up.

**Discussion:**

This randomised controlled trial is expected to provide insight into whether an educational video can improve the nutritional status of older adults following a surgical procedure and discharge from the hospital. The findings will be useful for assessing how videos offering nutritional advice to older adults who have undergone a surgical procedure can improve their health-related quality of life, reduce loss of function, prevent readmission to hospital and reduce healthcare costs.

**Trial registration:**

ClinicalTrials.gov NCT05950373. Registered on 11 July 2023.

## Introduction

Up to 50% of older adults are at nutritional risk during a hospital stay, and their nutritional status often deteriorates [1, 2]. Nutritional deterioration is a challenging healthcare issue, as timely intervention and consideration are required to mitigate the threat to health gains, preserve older adults’ independence in activities of daily living (ADL) and functioning and avoid readmissions [3]. Patients’ energy and protein intake from food is lower than needed during a hospital stay [4]. Furthermore, 20–45% of older adults are at nutritional risk when discharged from the hospital [5, 6]. Thus, the prevalence of nutritional deterioration after hospital discharge is a further challenge [7, 8]. A systematic review and meta-analysis reported the prevalence of nutritional deterioration in hospital, residential care and community settings to be 28% [95% CI 26.0–30.1] in patients ≥ 65 years of age from European countries [9], and the occurrence of nutritional deterioration among community-dwelling older adults in rural communities (9.9% [95% CI 4.5–16.8%]) is twice that of older adults in urban areas (5.7% [95% CI 4.2–7.3%]) [10]. Less access to family support and health services has been linked to higher malnutrition rates among those living in rural areas [10].

Surgical patients require sufficient energy and protein intake after surgical procedures to maintain muscle mass and prevent protein catabolism [11, 12]. The length of hospital stay (LOS) has decreased in recent decades [3]. Even severely ill patients are being admitted for shorter periods, and those with less severe conditions receive daytime care and outpatient treatment [3]. To improve the efficiency of inpatient surgery, many hospitals have introduced fast-track care pathways to reduce LOS for operations, including total hip replacements [13]. Fast-track regimes increase demands on patients to administer self-care after discharge [14]. Furthermore, hospitals are pressured to provide early discharge, releasing patients once they are medically stable [15]. This might result in discharge before patients’ nutritional intake and status are optimal.

Balancing cost-effectiveness and quality when providing care for older adults is challenging [16]. Person-centred care is often described as a model or philosophy of care that aims to acknowledge, involve and build upon the subjective experiences, needs, priorities, preferences and values of the person in need of care [16]. Person-centred care has been considered an appropriate approach to meet the challenges facing the healthcare system, but no consistent, agreed-upon parameters for providing such care exist [17]. A previous review found that person-centred care depends on [1] patient participation and involvement, [2] the patient–health professional relationship and [3] the care context [18].

The European Society for Clinical Nutrition and Metabolism (ESPEN) defines ‘malnutrition’ as the ‘deficiency, excess or imbalance of protein, energy and other nutrients, causing measurable adverse effects on tissues and function, and clinical outcome’ [19]. Although malnourished individuals can be over or undernourished, ‘malnutrition’ is often considered to be synonymous with ‘undernutrition’ [20]. The terms ‘undernutrition’ or ‘protein–energy malnutrition’ are further utilised to describe inadequate protein and energy intake or absorption [19]. Being at nutritional risk indicates that an individual is at risk of developing malnutrition or undernutrition. Disease-related malnutrition occurs due to diseases and disease states that entail the loss of body fat and muscle tissue [21]. Disease-related malnutrition is further connected to reduced dietary intake, increased nutrient loss, malabsorption or altered metabolic demands [20]. Older adults with a body mass index (BMI) lower than 24 are at nutritional risk [22]; therefore, it is recommended that older adults have a BMI between 24 and 29 [22].

Surgery can stimulate patients’ inflammatory, immune and metabolic responses, resulting in a hypermetabolic-catabolic state [23]. Despite interventions to reduce the metabolic response, such reactions can last from hours to days or even months, depending on the surgical invasiveness, anaesthesia used and postoperative complications [12]. Protein catabolism increases as a result of the stress response to surgery [24], which induces hypercortisolaemia, thus reducing protein synthesis and increasing protein breakdown [24]. The result is an outflow of amino acids from the skeletal muscle to provide amino acid precursors to support gluconeogenesis, immune function and wound healing [12]. Immobility that often accompanies major surgery can entail significant skeletal muscle loss, which is augmented by the muscle catabolism associated with the stress response to surgery [24]. In healthy individuals, muscle tissue loss begins to occur after as short a period as 48 h of inactivity, with significant loss within 5 days [24]. In older individuals, the rate of post-surgical atrophy is about 1% per day [25], with reductions of up to 18% in the quadriceps and hamstring of the operated leg within 6 weeks [24].

Furthermore, after total knee arthroplasty, older adults can experience up to a 14% reduction in muscle volume within 2 weeks [25]. Hip fracture patients may stay in a hypercatabolic state as a result of residual inflammatory syndrome for up to 3 months after surgery [26]. Consequently, malnutrition is often associated with prolonged rehabilitation, more frequent complications (e.g. infections), loss of ADL functions and higher morbidity and mortality [27, 28]. Malnutrition has also been seen as an independent predictor of low health-related quality of life [29], which can be measured by using the coherent, generic and easily administered 36-item Short Form Survey (SF-36) [30]. The Norwegian RAND 36-Item Short Form Health Survey (RAND 36) is a translated version of the SF 36 questionnaire. This Norwegian version has been used in previous studies and is considered valid and reliable [31, 32].

Nutritional intake is a behaviour that is guided by multiple factors. Three main factors have been identified:

i. Predisposing factors (e.g. knowledge, values, attitudes, experiences and enjoyment of eating)
ii. Enabling factors (e.g. ability to eat the food, such as cutting, chewing or swallowing; acute or chronic diseases; and access to buying and cooking)
iii. Reinforcing factors (e.g. spouses, relatives, healthcare staff and situational factors) [33, 34]

Healthy eating habits are relative and change due to people’s situations. Nutritional guidelines highlight the importance of increased energy and protein intake postoperatively and in the recovery phase, contrary to dietary recommendations for leaner products in the standard healthy elderly diet. Furthermore, food and drink intake during the hospital stay and the recovery phase afterwards might be compromised by several factors, including chewing or swallowing problems, sensory losses and anorexia, together with acute or chronic diseases. Therefore, a lack of knowledge of proper nutritional intake after surgery may compromise dietary intake and lead to nutritional deficiencies and malnutrition [35]. Older adults often have well-established eating habits [36]. Furthermore, they often lack knowledge of the need for increased protein and energy intake regardless of whether they are ill [37]. Surgery challenges patients’ nutritional behaviours and beliefs, requiring adjustments in the knowledge of relatives and healthcare professionals to support suitable nutritional choices.

Support from relatives is important [33, 34, 38], but misunderstanding what constitutes healthy food choices after surgery might lead relatives and neighbours to provide advice that negatively impacts older adults’ choices [33]. Previous malnutrition intervention studies focused on phone follow-up and face-to-face contact, which is resource-intensive for an already strained healthcare system [39]. In collaboration with a university librarian, we did not identify any studies that argued that education videos lack efficacy in nutrition education. According to our scientific literature search, the effectiveness of a health education video in improving the nutritional conditions of older adults after surgical treatment has not been confirmed.

Acquiring knowledge about their health can empower individuals to understand the necessary steps to take to prevent and mitigate the onset of disease and is therefore a fundamental requirement for self-care [40]. Educational videos provide relevant health information to patients and relatives and allow healthcare staff to translate and target information to the patient’s specific situation, preferences and context. Video-based educational interventions have been used to educate elderly patients about preventing foot ulcers, using ageing services and preventing falls at home [41–43]. However, no article has reported the effects of nutrition-related videos on the elderly after surgery, and there is a great need for person-centred care approaches for older adults, especially in out-of-hospital settings [44, 45].

Society and digital development have led to the digitisation of many services and activities. In Scandinavia, a Swedish study reported that 62.4% of adults over 66 use the Internet [46]. Other European studies found that approximately 49–55% of older adults use the Internet to varying degrees [47, 48]. Older adults often use the Internet or smartphones to search for information (e.g. health-related information or news), entertainment (e.g. videos or music) or to communicate with others [49, 50]. A precondition for using web-based health and social services is the ability to use and access the internet [51]. Digital healthcare can thus increase digital exclusion, as not all individuals are able to access digital solutions or possess the digital knowledge, skills or opportunities to benefit from their potential advantages. Nonetheless, older adults can positively use health technology to increase nutritional intake, given the necessary user education [52].

Inspired by the lack of digital videos focusing on the prevention and treatment of malnutrition in homebound older adults after discharge from hospitals, we developed such a video from 2021–2022. The development of the education video was followed by a 7-month single-centre, two-arm feasibility study conducted in a rural area in northern Norway from May 2022 to January 2023 [53]. The feasibility study aimed to determine the feasibility of recruitment as well as data collection in accordance with a future full-scale intervention study where the intervention group received access to a 6-min-long education video, and the control group only accessed standard care [53]. The feasibility study showed the need for a larger full-scale study with some adjustments related to the follow-up after receiving the video. Based on the results from the feasibility study [53], it was not necessary to modify the video before a full-scale trial.

### Aim of the study and research question

This study aims to develop and test the effectiveness of an educational video to prevent loss of health-related quality of life among live-at-home older adults following discharge from the hospital after surgical treatment. For the purpose of this study, older adults are defined as persons who are 65 years of age or older.

The research question of this study is as follows: How does a digital teaching video affect the nutritional choices and intake of older adults living at home, aiming to maintain nutritional status, functioning and health-related quality of life during their recovery at home after post-surgery discharge?

### Hypotheses

This study hypothesises that translating nutritional guideline recommendations regarding the intake of energy and protein using a digital medium for older adults aged 65 years and older will stimulate them to increase their intake of energy and protein in the recovery phase after discharge from the hospital following a surgical procedure. This will prevent loss of weight, loss of muscle mass and declines in health-related quality of life.

To verify the hypothesis, the following data will be collected: The participants in the inclusion group will be measured on their knowledge of highly recommended food choices, maintenance or increase of their BMI, mid-arm circumference (MAC), triceps skinfold thickness (TSF), mid-arm muscle circumference (MAMC), handgrip strength, knowledge of nutritional choices and health-related quality of life 3 months after discharge from hospital.

## Methods

### Study design

This study is designed as an experimental single-centred, two-arm parallel group randomised controlled trial over 11 months with a 1:1 allocation ratio.

### Study setting

The study will be conducted at a regional hospital in a rural part of Northern Norway. Participants will be recruited from 3 surgical departments: the orthopaedic department, the urology and vascular/thoracic surgery department and the gastro, gynaecological, breast and surgical endocrine department. The hospital serves a broad range of mental and somatic healthcare and substance abuse patients from 20 municipalities in the region. The annual budget of the hospital is approximately 5 billion Norwegian kroner. The number of employees is about 4800. Due to logistical reasons affecting the project, which will be carried out in a rural area, patients eligible for inclusion in the study must live in 1 of 9 predetermined municipalities closest to the hospital and the location of the research team. The 9 chosen municipalities have between 1000 and 53,000 inhabitants. The Norwegian healthcare system (both primary and secondary) is publicly funded and ensures all residents with free access to care.

### Participants and recruitment strategy

Surgical patients over 65 years of age will be eligible to participate in the study if they meet the following criteria:

1. They are inpatients.
2. Their home address is one of the municipalities affiliated with the selected hospital.
3. They live at home and are due to return home after discharge directly from hospital or after a short-term training stay at a training centre before returning home.
4. They can read and understand Norwegian.
5. They have consent competence.
6. They have a mobile phone or PC/tablet able to connect to the Internet.
7. They have a body mass index < 24.

Following guidelines by Helsedirektoratet [54] and using NRS 2002 [55] for nutrient screening, the study will be based on the premise that all bedridden older adults undergo nutrition screening during their hospital stay and therefore have a known BMI. Patients receiving only liquid diets, such as tube feeding or intravenous nutritional support, and terminally ill patients will be excluded from the study.

The first author will have access to the department’s record system and will be responsible for informing and obtaining consent from eligible patients. The first author will enter the data into a spreadsheet and assign the participants to the intervention or control groups after the randomisation has been done. Furthermore, a trained external data collector who is not involved in the project will perform all the measurements.

### Sample size

We base our calculation of the sample size on the effect estimates of the primary outcome of the combined physical scale from the feasibility study, as estimates were not available in the literature [53]. Alpha was set at 0.05, and the beta was set at 0.20. The estimated effect size was calculated to be 7 points on a scale ranging from 0 to 100, and the standard deviation of the population outcome was 18. The patients will be distributed with 50% in the control and intervention groups. Using the sample size-means calculator [56], the necessary sample size was calculated to be 104 in each of the 2 groups. Based on the feasibility study [53], we estimated a drop-out rate of 32%. Therefore, we need to recruit 138 participants for each group.

### Randomisation

Complete randomisation will be achieved using the free software program RANDOM.ORG [57]. The first author will use the program to allocate participants to the inclusion group or the control group randomly. The study will be blinded to the care providers and data collectors, including the external data collector who will perform the outcome assessors and the statistician (investigator). A code will be allocated to each patient and not revealed before the analysis has been completed. Once the analysis has been completed, the group to which participants had been allocated will be revealed. Due to the nature of this study, where the participants will be aware whether they will have access to the educational video, it is not possible to achieve blinding for the participants.

### Intervention

Participants in the intervention group will be given access to a 6-min educational video 5 days after discharge from the hospital. This will allow the participants to become settled at home or at a training centre to ensure their receptiveness to the intervention. The educational video will present dietary recommendations from the *Kostholdshåndboken* (English: *The Diet Handbook*) published by Helsedirektoratet [58]. The dietary guidelines recommend that malnourished older adults increase their energy and protein intake and provide examples of how older adults can adapt and integrate the recommendations into their preferences and daily mealtime routines [58]. As the participants in the intervention group will be required to access the educational video via their email or smartphone, the video will be made accessible individually through a link sent out by the first author. Through the video, the participants will be encouraged to watch the video several times, with, for example, their relatives or other health personnel. Since it will not be possible to personalise the intervention to each participant, the video information on food and nutrition is based on the following general principles:

1. The information should be simple and correct, according to Contento and Koch [59]; this means keeping the words simple, since the field of nutrition can be full of technical jargon.
2. The information is presented using the active voice rather than the passive voice, since this makes the text more personal [59].
3. The length of sentences varies but is generally kept short [59]. The text of the education manual has undergone several ‘translations’ and revisions to make it easier for people with different assumptions and needs for information to understand. To prevent confusion among patients and strengthen the study’s criterion validity, we collaborated with a nutritionist who provided daily information to the user group to ensure we used precise words and expressions. Furthermore, the face validity of the educational video was confirmed by two bed-ridden patients in the user group who read the script and gave oral and written feedback on its contents. The intervention patients in the feasibility study did not have problems understanding the content, and significant changes in nutritional intake were observed.

In making the video, we used visual effects and pictures of different products so that participants would be able to imagine other food options more easily. Product images were obtained following current legislation, and there are no conflicts of interest or collaboration between the producers of the product images and the study’s authors.

The control group will have no access to the video and will not receive other intervention dietary advice in relation to this study. The selected hospital performs nutritional screening of patients in connection with admission as a part of the standard procedure at the hospital but does not provide nutritional information brochures or similar information upon discharge. However, as a standard procedure at the selected hospital, malnourished patients can receive advice or follow-up from healthcare professionals such as nutritionists on request, regardless of the intervention. No concurrent treatment or intervention will take place during the trial.

To strengthen retention, the participants in the intervention group will get a text message each week for the first 3 weeks after discharge and be contacted by phone to reduce technical problems opening the link, etc.

At follow-up after 3 months, the participants in the intervention group will be asked if they have seen the video and if they can remember how many times they have seen it. At the end of the intervention period, the authors will meet to review that both participants in the intervention group have been given access to the intervention as planned and that the intervention has not been given to the control group. The education video will not be modified during the course of the study.

### Measurements

**Fig. 1:**
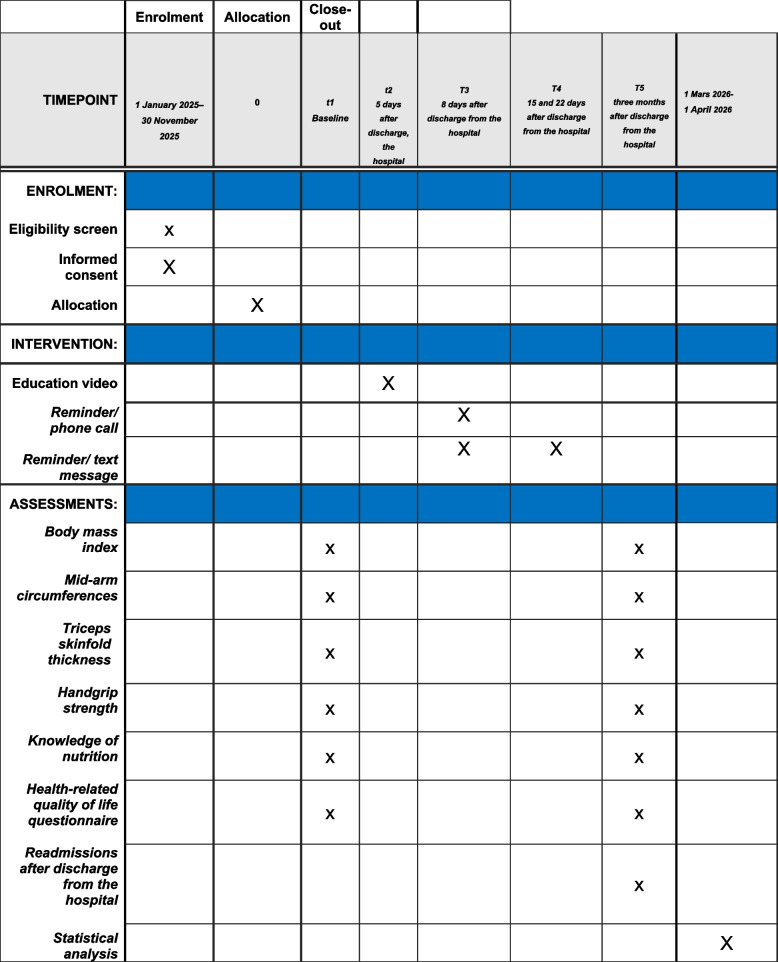
SPIRIT figure

The measurements will mainly focus on basic information, health-related quality of life, body composition measurements and readmission to hospital. The same externally trained data collector will carry out all measurements during the patients’ hospital stays and 3 months after discharge. We will register the number of readmissions within 3 months after discharge from the hospital. Figure 1 shows the schedule for enrolment, the intervention and the assessment of the trial according to the SPIRIT template.

### Demographic and clinical information collection

The patients’ demographic information, including name, age, email address, phone number, level of education and need for home care, will be collected at the baseline in the hospital. Information on LOS and the number of readmissions will be collected from hospital records 3 months after discharge. This trial will not involve the collection of biological specimens for storage.

### Primary outcome

#### Health-related quality of life

Health-related quality of life will be assessed using the 36-item Medical Outcomes Study Short Form Questionnaire (RAND 36). The RAND 36 is grouped into eight health concepts using multi-item subscales that are combined into two main scales summarising physical and mental health. The physical health component correlates mainly with the subscales of bodily pain, physical functioning and role limitations on physical scales [60]. The mental health component correlates mainly with the three subscales of social functioning, mental health and role limitations on emotional scales [60]. The two subscales of general health and vitality have noteworthy correlations with both the mental and physical health components [60]. The participants’ answers will then be analysed and given a score following the recommendations of the RAND Corporation [61].

### Secondary outcome

#### Body mass index

The participants will be weighed upon admission to the hospital. Their weight will be measured in the morning, when they are wearing light clothes without shoes and using the electronic Withings Body Cardio WBS04; their weight will be recorded to the nearest 0.1 kg. To be sure that only the Withings Body Cardio weight will be used in all weight measurements, the use of bed weights will be prohibited during this project. If participants cannot stand on the scale, weight data will not be collected. Height will be estimated based on forearm length, counting the ulna length between the point of the elbow and the midpoint of the prominent bone on the wrist. This value will be compared to the values in a standardised high conversion chart from RxKinetics [62] and recorded to the nearest 0.5 cm. BMI will then be calculated based on participants’ weight (kg) divided by the square of their height (m) [63]. Values < 24 kg/m^2^ will indicate malnutrition.

#### The mid-arm circumference

The MAC will be measured using the midpoint between the olecranon and the acromion, with the arm extended, the muscles relaxed and the palm facing the thigh. The measurement will be recorded in centimetres, using the left hand. The participants will be classified as malnourished if the MAC is less than 24 cm and well-nourished if it is more than 24 cm [64].

#### Triceps skinfold thickness

The TSF will be measured to estimate the fat mass in millimetres. A VirtuFits digital fat calliper will be used to measure the skinfold of the triceps of the left arm. The test will be repeated three times, and the average will be recorded.

#### The mid-arm muscle circumference

The MAMC will be calculated according to the following formula: MAMC (cm) = MAC (cm) − (3.14 × TFS × 0.1). Guided by Symreng [65], malnutrition will be indicated for women up to 79 years of age if the arm muscle circumference is less than 19 cm and for women over 79 years if the arm muscle circumference is less than 18 cm. Malnutrition will be indicated for men up to 79 years if the arm muscle circumference is less than 23 cm and for men over 79 years if the arm muscle circumference is less than 21 cm.

#### Handgrip strength

Handgrip strength will be measured using the Saehan Hydraulic Hand Dynometer and recorded to the nearest 0.5 kg for both the dominant and non-dominant hands. Following the manufacturer’s recommendations, the participant will sit with the shoulder adducted and naturally rotated, the elbow flexed at 90° and the forearm and wrist in a neutral position. The test will be repeated three times for each arm, and the average value for each arm will be recorded.

#### Knowledge of nutrition

Participants’ nutritional knowledge will be measured by questions asking them what they think would provide them with the most protein and energy among different kinds of food, such as apples, vegetables, meat, oat crackers and dairy products. For example, one of the questions is as follows: ‘Which of the following food categories do you believe is best for you to consume to ensure a good start to your rehabilitation: dairy products with little or a lot of fat?’ The inclusion group will be able to find answers to the questions through the educational video.

#### Readmissions after discharge from the hospital

Readmissions to the hospital will be measured for up to 3 months after discharge by each department’s internal data collector, who will have access to the department’s record system.

### Data management and monitoring

#### Data analysis

For statistical analysis, SPSS version 28.01 (IBM Corp., Armonk, NY, USA) will be used. The statistical analysis plan will be based on the principles of the intention-to-treat approach. All ratio–interval scaled data will be tested for distribution using the *F*-test. When normally distributed, parametric statistical methods will be applied, such as Student’s *t*-test and the one-way analysis of variance (ANOVA). The data will be presented through means, standard deviation (SD) and 95% confidence limits. Normal or ordinal data will be presented as absolute numbers and frequencies. Differences will be determined using non-parametric tests (e.g. the *X*^2^-test, Mann–Whitney test and rank-sum test). The data from the RAND 36 questionnaire will be decoded using the SF-36 ordinal subscales and will be transformed into a ratio scale using the syntax SF-36 manual. The results will be presented from each of the eight subscales and the two main scales. The reliability of the SF-36 scales will be calculated using Cronbach’s alpha. When the data analysis is complete, the code regarding participants’ allocation to either the intervention or control groups will be revealed. Data will be collected as long as patients are included in the study. If patients withdraw their consent, data collection will stop. Deviation from the protocol will be registered and reported accordingly. Concerning post-assignment attrition, we will conduct an attrition analysis based on variables such as the participants’ gender, age, education level, marital status and help from home care. The attrition analysis will thus reveal any systematic loss at follow-up and identify whether there are participants with a higher likelihood of attrition.

#### Monitoring

The first author will continuously monitor the study in relation to registration. Specifically, (1) data will be exhaustively registered, (2) the number of potential participants who refuse to participate will be tracked, (3) participants who have viewed the education video will be contacted 1 week after they have received the video to check that they have been given access to it and (4) participants and their relatives will be contacted to ensure they have received information on how to contact the project management group in the event that they have questions or unwanted incidents occur.

Furthermore, a steering committee will be responsible for providing support for the study. Members of the steering committee will be from Nord University, leaders from the involved wards at the hospital and a user representative. The primary author will be the secretary for the steering group. The steering committee will meet at least monthly and receive reports of the study’s progress each week.

The first author will inform the steering committee if changes to the protocol occur, and any deviations from the protocol will be fully documented. Changes in the protocol will also be updated in the clinical trial registry. Since the research will be done at only one hospital and this is a low-risk intervention, a coordinating centre, project management group or data monitoring committee will not be needed.

#### Adverse event reporting and potential harm

To our knowledge, there is no evidence of adverse or serious events resulting from the intervention. There is no anticipated harm and compensation for trial participation. Still, we are aware that the participants belong to a vulnerable elderly population, and they will receive contact information from the first author and be encouraged to make contact if unwanted incidents occur. Supposing an unexpected serious event should nevertheless occur, this will be reported to the relevant regulatory bodies (sponsor and hospital) as required, and such reports will indicate expectedness, seriousness, severity and causality within one working day following the project management group’s awareness. Interim analysis and stopping rules are not needed because this is a low-risk intervention.

## Discussion

The combination of an increasing number of older adults and short hospitalisation times will result in an increased need for early rehabilitation after hospitalisation. To our knowledge, this study is the first to investigate the effectiveness of an educational video designed to improve patients’ knowledge to ensure they intake a sufficient amount of energy and protein at home following a surgical procedure and hospitalisation. The results will yield evidence on how educational videos can prevent disease-related malnutrition, preserve older adults’ functionality after a hospital stay and reduce healthcare costs. Furthermore, digital health solutions can help alleviate staff shortages.

To ensure that the study addresses its specified purpose and hypotheses, validated measurement tools will be used. An earlier systematic review and meta-analysis found that nutritional intake can improve physical performance outcomes (e.g. handgrip strength) in older adults who were already affected by specific medical conditions or by sarcopenia or frailty [66]. Maintaining internal validity can be challenging, as body composition measurements among older adults are difficult to record, and the interpretation of the data can be more problematic and prone to error [67]. The method used to measure body composition should be convenient and easy to implement. It can be difficult to measure the height of patients with fractures when using the method of having them stand up against a wall. Therefore, the participants’ heights will be estimated based on the length of their forearms because this is the most convenient method for the participants that can simultaneously ensure validity, in contrast to patients self-reporting their height.

Skinfold measurements are considered a valid method for assessing a person’s body fat [67]. However, such measurements can be challenging to perform, as the difference between adipose tissue and muscle is more difficult to palpate in older adults [67]. It can also be hard to measure bedridden patients. Therefore, we will enlist trained personnel in the performance of measurements.

Methodological quality control is crucial in ensuring the repeatability of results. In this trial, quality control will be strengthened in two ways. First, we have developed rigorous inclusion and exclusion criteria. The data collector in each department will further discuss with the first author which patients can be included in the study. Second, we plan to have the same person perform the measurements at the hospital and 3 months after hospital admission. The research team will test this person on their skillset three times during the data collection period to ensure that the measurements will be taken correctly.

Since all intervention participants will view the same educational video, it is impossible to allow for major individual considerations. Thus, linguistic considerations and nutritional recommendations will be based on a standardised health guide. According to Halse [68], it is challenging to find a good balance when information is being delivered to a large group of individuals with different needs. Our study will focus on older adults, which is not a homogeneous group. Thus, making a video that meets every participant’s needs would be impossible. The educational video is, therefore, based on general guidelines. Using the Norwegian *Kostholdshåndboken* (English: *The Diet Handbook*) to increase each patient’s knowledge will increase the study’s content validity. To ensure that the participants will understand the language and content of the video, the script was first face-validated by two older adults who satisfied the inclusion criteria and second through the feasibility study that led to this trial.

Self-management programmes are designed to help patients master tasks related to their diseases and increase their confidence and ability to control troublesome health symptoms [69]. Health information technology can empower patients to take ownership of their health and treatment and enable healthcare providers to deliver better person-centred care [70]. An educational video can be viewed multiple times, anytime and anywhere, as is convenient for the individual. We may therefore argue that it can be more person-centred than, for example, only providing individuals with information right after an operation, while the patient has to be discharged quickly. We further argue that the educational video developed for this study can be a starting point for a more person-centred relationship between older adults and healthcare personnel, since the relationship can become more focused on individuals’ requests. However, a limitation of this intervention is that it will exclusively address those patients with internet access and the necessary health literacy skills to use and understand the video content or those who can be helped by healthcare personnel or significant others to access the video and implement the information in the everyday lives. For other patient groups, other health interventions might be more relevant. Therefore, in a pressured healthcare system, a health-related educational video can be a solution for some patients but not necessarily a routine intervention for all.

This study will increase patients’, relatives’ and healthcare staff’s knowledge of the need for a sufficient intake of energy and protein after surgery and inform them that the recommendations for healthy eating are different in a period of convalescence. This will contribute to preventing postoperative malnutrition, thus reducing costs for healthcare organisations related to complications following surgery, mitigating loss of function and preventing reductions in patients’ health-related quality of life.

## Trial status

The current protocol version registered on ClinicalTrials.gov is dated 2 April 2024. Data recruitment is planned to commence on January 1, 2025, and run for 11 months, up to and including November 30, 2025.

This clinical trial protocol was designed following the Standard Protocol Items: Recommendations for Interventional Trials (SPIRIT) as evidenced by the checklist. The time points for enrolment, intervention and assessment are shown in Fig. 1.

## Data Availability

All investigators involved in the study will have full access to the data. The results of the study will be presented at conferences, through scientific publications and in academic articles. The participant information materials and informed consent form will be available from the corresponding author upon reasonable request. Any data required to support the protocol can be supplied on request. Data will be made available in accordance with Nord University’s current guidelines.

## Abbreviations

LOS: Length of hospital stay
BMI: Body mass index
MAC: Mid-arm circumference
MAMC: Mid-arm muscle circumference
RAND 36: RAND 36-Item Short Form Health Survey
SF-36: 36-Item Short Form Survey
TSF: Triceps skinfold thickness
